# Effects of a High Intensity Interval Training (HIIT) Program on Anthropomorphic and Cardiometabolic Variables in School Children with Overweight and Obesity

**DOI:** 10.3390/children10020317

**Published:** 2023-02-07

**Authors:** José Miguel Espinoza Silva, Pedro Ángel Latorre Román, José Carlos Cabrera Linares, Juan A. Párraga Montilla, Cristian Martínez Salazar

**Affiliations:** 1 Departamento de Educación Física, Deportes y Recreación, Universidad de La Frontera, Temuco 1145, Chile; 2 Departamento de Didáctica de la Expresión Musical, Plástica y Corporal, Universidad de Jaén, 23071 Jaen, Spain

**Keywords:** HIIT, weight status, schoolchildren, cardiometabolic risk

## Abstract

The aim of this study was to assess the effects of a high intensity interval training (HIIT) program on anthropomorphic and cardiometabolic variables in schoolchildren with overweight and obesity. A total of 443 schoolchildren (age: 6.37 ± 0.65 years) took part in this study. The experimental group (EG; n = 295; age = 6.40 ± 0.64 years) was compound with children with overweight and obesity, whereas children with normoweight were included in the control group (CG; n = 148; 6.31 ± 0.67 years). The EG performed a training program based on HIIT two times per week for 28 weeks (56 sessions), whilst the CG performed their habitual physical education classes based on the national curriculum. Body mass index (BMI), waist circumference, body fat, ∑ 4 skinfold fat, waist to height ratio, waist circumference, and cardiometabolic risk were measured. The dependent variables were analysed by two-way analysis of covariance (ANCOVA 2 × 2). To analyse the percentage differences between groups, the chi-square test was used. *P*-value was set at *p* < 0.05. Significant differences were found in the EG in BMI, waist circumference, body fat, ∑ 4 skinfold fat, and waist to height ratio. In conclusion, an HIIT training program can be an effective tool for improving anthropomorphic variables and reducing cardiometabolic risk in schoolchildren with overweight and obesity.

## 1. Introduction

Physical activity (PA) classes at school are considered the ideal opportunity to teach children to incorporate PA into their habits for a healthy life [1]. The practice of PA to improve health is well documented, and the scientific literature has identified a range of benefits associated with this practice by children [2]. Nowadays, the daily PA recommended for schoolchildren is 30 min of moderate to vigorous intensity per day in a physical education (PE) class and 20 additional minutes in breaks, when it is assumed that the children will be active [3]. Data collected in Chile by the National Survey of “Physical Activity and Sports Habits” in 2019, in the population aged between 5 and 17 years, indicated that 48.4% of this population was inactive [4]. In addition, a previous National Health survey carried out in 2016–2017 by the Chilean Health Ministry showed that only 12.5% of the general population perform PA [5].

The 2019 Nutritional Map of Chile showed that the obesity (OB) percentage in schoolchildren (pre-school and primary education) was 52.1%. However, a different situation prevails for severe OB, which has been increasing by 0.3 percentage points (pp) per year compared with a study carried out in 2018. According to the report mentioned above, there are regions in Chile that have maintained a steady increase in the percentage of obesity for ten consecutive years. For instance, in the Araucanía Region (home of the schoolchildren studied in the present research), the percentage of children with OB has risen from 45% to 69.3% in the last ten years [6]. In 2020, the National School Support and Grants Authority (JUNAEB) evaluated the nutritional state of children during the COVID-19 pandemic. The results showed an increase of 2 pp in the total prevalence of OB in relation to the survey carried out in 2019. Moreover, an important finding was that the schoolchildren’s height growth had been retarded by 1.6 pp compared to the previous year [7]. The causes of child OB in Chile can be considered multifactorial, two of the most important factors being unhealthy eating habits and physical inactivity [8].

The time assigned to PE in the Chilean national curriculum is only 2 h/week, which goes against the recommendations of World Health Organization [1], since a large percentage of the Chilean population never does any PA outside these periods. Over the years, this has led to an alarming increase in levels of overweight (OW) and OB, implying a risk of developing various non-transmissible diseases early in life, resulting in an impact on the health care system [9]. Previous research concluded that traditional training programs, like cyclical activities (walking, jogging, treadmills, ergonometric bicycle, etc.), which are usually performed by adults to control anthropomorphic and metabolic indicators, are considered boring and unattractive by children [10]. Consequently, they do not encourage the regular practice of PA [11,12,13,14]. To solve these problems (OB and OW) and avoid the unattractive training method (i.e., cyclical activities), high intensity interval training (HIIT) has been identified as an effective training method in the school population [15,16].

HIIT is a training method which combines brief bursts of vigorous activity (exercises executed above the lactate threshold) interspersed with short recovery periods performed at low intensity [17]. HIIT has also been reported to play an important role in physical composition and metabolic risk [18]. Furthermore, HIIT is a time-efficient training method since it requires less training volume. It also provides greater health improvements (e.g., cardiometabolic and body composition parameters) in children and adolescents compared with traditional training programs [19]. Consequently, there has been growing interest in the use of HIIT in children since it is more enjoyable than other training methods. Moreover, HIIT has shown to have faster results when the goal is to recover children’s normal weight, since desertion from the training program is lower than from other training programs [20]. Previous research has concluded that based on children’s perceptions of OW, and their evaluation of different PA programs, HIIT was selected as the most enjoyable [21]. This should be taken into account when selecting a PA program for primary school children; therefore, HIIT could be a very influential factor for long term adherence to physical exercise [21]. Moreover, a long-term HIIT program can be an effective method for reducing OW and OB in children [22]; however, it is necessary to adapt the characteristics of HIIT to children’s needs, being high intensity games are an effective way of improving health-related parameters in schoolchildren. Previous research concluded that games are more enjoyable and would generate a greater motivation to practice PA in children with OW or OB [23].

Therefore, the aim of this study was to assess the effects of a long-term HIIT training program in anthropomorphic and cardiometabolic variables in schoolchildren with OW and OB.

## 2. Method

### 2.1. Participants

A total of 443 schoolchildren (age: 6.37 ± 0.65 years) took part in this study. The experimental group (EG; n = 295; age = 6.40 ± 0.64 years) was composed of OW and OB children; the control group (CG; n = 148; age = 6.31 ± 0.67 years) included normoweight children. The children were selected from six schools in the city of Temuco (Chile). The inclusion criteria were (a) being in their first or second year of primary education; (b) demonstrating an absence of physical and intellectual disabilities; (c) having a medical certificate permitting physical exercise. The parents/guardians voluntarily signed informed consent forms allowing the participation of their children in this study.

The study was carried out in accordance with the principles of the Declaration of Helsinki (2013) and follows the European Guidelines on Good Clinical Practices (111/3976/88 of July 1990) as specified in the Spanish legal framework for clinical investigation in humans (Royal Decree 561/1993 on Clinical Trials). The study was approved by the Ethics Committee of Universidad de Jaén (Jaén, Spain).

### 2.2. Materials and Procedures

#### 2.2.1. Anthropometric Measurement

The body mass (kg) was measured using a portable scale (TANITA, model Scale Plus UM -028, Tokyo, Japan). The children were instructed to wear light clothing and they were weighed barefoot. Their height (cm) was measured with a Seca^®^ stadiometer, model 214, Hamburg, Germany).

#### 2.2.2. Body Mass Index (BMI)

The BMI (%), defined as the ratio between the bodyweight and the square of the height (kg/m^2^), was used to estimate the degree of OW and OB. The weight status of the participants was determined by percentile (p) according to the following criteria: OW—BMI between p 85 < p 95; OB—BMI > p 95 [24]. The waist circumference (WC) was measured with a tape measure (Seca^®^, model 201, Hamburg, Germany) at the height of the navel [25].

The percentage of body fat (BF) was evaluated from bicipital, tricipital, subscapular, and suprailiac measurements using Slaughter’s equation [26]. The assessment was carried out with a skinfold caliper model 102-602 L (Minneapolis, MN, USA).

#### 2.2.3. Assessment of Cardiometabolic Risk

Cardiometabolic Risk (CR) was calculated from the waist to height ratio (WHtR). The WHtR was obtained by dividing the WC by the height of the participants, and this value was used to estimate the accumulation of fat in the central region of the body. Previous studies have shown the effectiveness of the WHtR in detecting metabolic alterations in the paediatric population in general (of either sex and any age) [27], using a cut-off point greater to or equal than 0.5 [28].

To measure the blood pressure (BP, diastolic and systolic), a digital portable device was used (OMRON^®^ model HEM 7114; Lake Forest, IL, USA).

Aerobic capacity was assessed with the 6 min walking test (6MWT) adapted for children. This test assesses the distance that the participant is able to walk in 6 min [29].

### 2.3. Intervention

Schoolchildren with OW and OB performed a HIIT program for 28 weeks (two sessions per week) during term-time from April to October. They performed a total of 56 sessions, each session lasting 40–50 min. The CG attended their normal PE classes based on the national curriculum, which usually include low intensity activities and little incentive to effort [30]. The PE classes included activities for the various curriculum topics, such as motor skills; a healthy, active life; safety; fair play and leadership; health and attitudes [31]. During each PE class, the children with OW and OB (EG) were separated from the normal-weight children and performed the HIIT program separately. Each session was conducted by a professional PE teacher who participated voluntarily in the study. This procedure was repeated in all schools that decided to join in our research.

The main strategies of the study training program (which incorporated the principles of HIIT) were high intensity games, ludic activities (i.e., traditional games, activities selected by the children and performed with high intensity in order to reach the HIIT principles; they also included outdoor activities in natural spaces for chase games and races, and games to promote basic motor skills like jumping. These strategies are similar to those reported in previous research [32].

Each session began with 5 min of warming-up activity, with chase games and joint mobility exercises, and ended with 5 min of calm-down activities. The main part of the session was divided into two blocks: in the first block, lasting 15–20 min, the participants performed high intensity activities for between 30 s and 1 min, followed by a rest time (1 to 2 min); the second block had a similar duration (15–20 min), and consisted of lower intensity activities for 4 min, followed by 1 to 2 min recovery time (3–4 sets). To establish the time and intensity of the activities, we followed recent suggestions for HIIT in schools [14]. The intensity of each activity was estimated with reference to the 10-point modified Borg scale for children, considering high intensity activities to be those performed at over 8 points on this scale [33,34].

### 2.4. Statistical Analyses

The data were analysed with SPSS v19 for Windows (SPSS Inc., Chicago, IL, USA) and the level of significance was set at α = 95%. The data were reported as descriptive statistics, with means, standard deviation (SD), and percentages. Normal distribution and homogeneity of the data for analysis were tested using the Kolmogorov–Smirnov and Levene’s tests, respectively. The dependent variables were analysed by two-way analysis of covariance (ANCOVA 2 × 2) with repeated measures (one group per measure). To analyse the percentage differences between groups, the chi-square test was used. The sizes of effect for the differences between groups were expressed as Cohen’s d and reported as: trivial (<0.2), small (0.2–0.49), moderate (0.5–0.79), and large (≥0.8) [35].

## 3. Results

Table 1 shows the anthropomorphic data and factors that have an influence in CR. In relation to BMI, significant differences were found in the EG after the intervention in BMI, while the CG did not show significant changes in this variable. Regarding WC, the CG showed an increase in values in this variable. Similar values were found in BF, since a reduction was also found in the EG in this variable *p* < 0.001. In addition, the EG showed a significant reduction in ∑ 4 skinfold fat. Finally, BP and RHR showed no significant changes (*p* ≥ 0.05).

**Figure 1:**
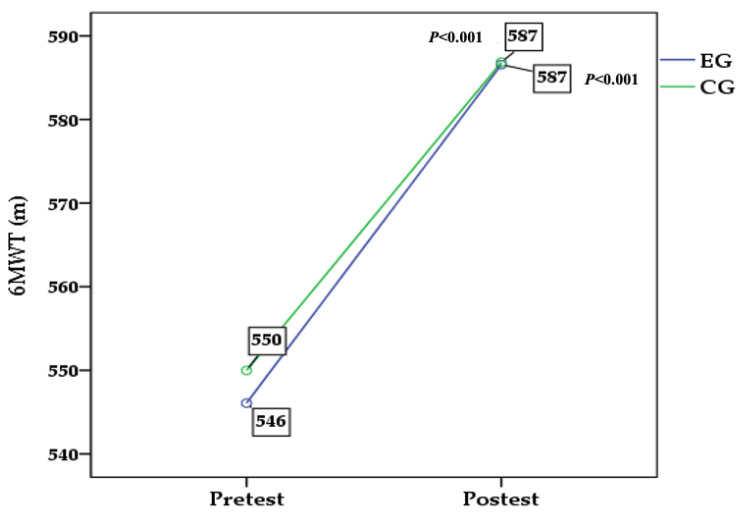
Performance in 6MWT before (pre-test) and after the intervention (post-test).

In relation to the distance performed in the 6MWT, both groups improved from their initial values.; however, no differences were found between groups. Figure 1 shows the performance in 6MWT.

**Figure 2:**
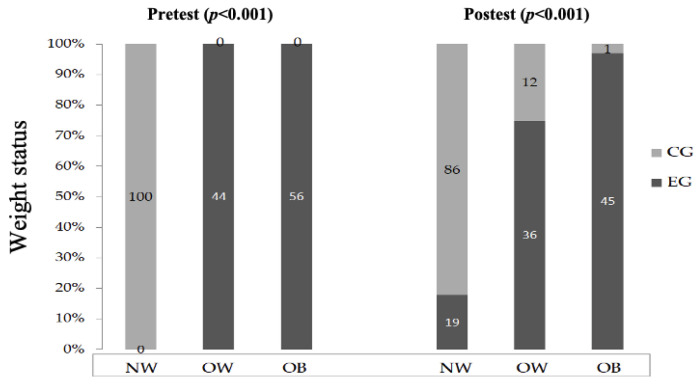
Weight status before (pre-test) and after (post-test) training program. EG: experimental group; CG: control group; NW: normoweight; OW: overweight; OB: obese.

The participants’ weight status before (pre-test) and after (post-test) training program according to the Chilean state are shown in Figure 2. EG, reduced significantly the percentages of overweight and obesity after the intervention.

## 4. Discussion

The aim of this study was to assess the effects of an HIIT program on anthropomorphic and cardiometabolic variables in schoolchildren with OW and OB. The main findings are that the HIIT training program had a positive effect on BMI, % BF, ∑ 4 skinfold, as the EG showed a reduction from the initial values of all these parameters. Consequently, the number of schoolchildren with OW and OB was reduced after the HIIT intervention.

In relation to anthropometric parameters, it should be noted that weight (increase or decrease) is difficult to control since it depends on several factors such as the family’s food habits, number of hours/weeks practicing PA, number of sedentary hours, and hours of sleep [36]. Despite these uncertainties, the EG showed a reduction of BMI. Our results are in concordance with previous research [22] which obtained a reduction in BMI among other health-related variables with a training program of a similar duration to ours. In addition, our results are in agreement with those reported by Blüher et al. [37], which concluded that an HIIT program had a positive effect on BMI and cardiometabolic health, although these authors conducted their research in an adolescent population. Nevertheless, the findings of the current study do not support the research carried out by Martinez et al. [38], since those authors did not find significant changes in BMI after their intervention in schoolchildren. The disagreement between studies could be due to the duration of their program, which was only 12 weeks—less than half the time of our intervention. Hence, 12 weeks may therefore not be enough time to obtain an effect on BMI in children.

Additionally, we assessed the WHtR to obtain more accurate information about the body composition of the participants. WHtR gives us important information about the location of BF. Moreover, it shows the degree of abdominal OB, which is a useful predictor of CR in children and adolescents [39]. Furthermore, WHtR allows changes to be tracked over the years in children and adolescents [40]. Notice that a 0.50 cut-off point is widely used to identify CR. However, a recent meta-analysis concluded that a simple cut-off of 0.50 can be inappropriate for predicting CR [41]. Consequently, it should be combined with other measurements to get a proper assessment of body composition in children.

Furthermore, it should be noted that the EG a reduction of ∑ 4 skinfold fat. This is an important finding in the present study due to the frequent accumulation of fat in subcutaneous deposits, which implies an increase in the risk of cardiovascular diseases in children and adults [42]. Our findings are in agreement with the study carried out previously by Lau et al. [43]. The authors concluded that an HIIT intervention improved body composition in OW children, despite the differences in protocol, since their HIIT program was conducted through an interval run test, namely the yo-yo test [42]. Nevertheless, our findings contradict those of Thivel et al. [44], since after a long-term PA intervention (6 months), they did not observe any improvement in body composition. A possible explanation for these results is that their study protocol was based on increasing PA time and minimizing inactivity, instead of HIIT as in the present research.

On the other hand, BP is recognised as one of the most important factors of CR in children [45]. Moreover, high BP levels in children have been associated with a higher risk of hypertension in later stages of life (e.g., adulthood) [46]. Consequently, it is necessary to reduce high BP values in children to avoid and prevent cardiovascular diseases. In the present research, the participants showed small changes in both groups; however, their BP values can be expressed in terms of normal values according to the reference values in these ages [47]. These results match those observed in a recent meta-analysis [48]. Moreover, these results agree with the systematic review carried out by Bauer et al. [49]. In their systematic review the authors obtained minimal improvements in cardiovascular fitness parameters (i.e., maximal oxygen up-take, maximal heart rate, diastolic, and systolic BP). A possible explanation for the minimal changes may be that the initial BP values in the participants were normal at the moment of the assessment. Hence, small changes can be expected.

In the 6MWT, both groups improved from their initial values, covering greater distances in the post-intervention assessment since the distances achieved were slightly above the reference values for their age [50]. The findings observed in this study agree with those of del Corral et al. [51], who also found an improvement between pre- and post- 6MWT measurements (4 months between assessments) although no training program was conducted. Also, the values obtained in our study are in concordance with the reference values exposed in the Chilean schoolchildren population [52]. The improvement in this test can be explained by the training program performed, and with factors related with the children’s growth (i.e., age, weight, and height) [51]. Consequently, these factors should be kept in mind when the 6MWT is conducted in children.

Regarding CR, our results showed that the EG reduced their CR values. The present findings seem to be consistent with previous research in which the authors concluded that 28 weeks of HIIT intervention have a positive effect on health-related variables in school-children [53]. This also agrees with the study conducted by Lambrick et al. [54]; their findings support that an intervention based on high intensity games induces positive changes in physiological and anthropometric indices in children with OB. The agreement of our findings with these results, despite the difference in duration, could result from the fact that it is not clear which HIIT protocol induces the greatest health benefit in children’s health [55]. However, it seems clear that an HIIT program will produce greater improvements than a low intensity training program in children [56].

### Strengths and Limitations

Our study has some limitations that have to be mentioned: (1) although the intervention sessions were based on high intensity activities, we did not measure the intensity of the activity objectively with portable devices such smartwatches or similar; (2) we did not control the participants’ diet during the intervention program; (3) we did not control the extracurricular PA that the participants carried out during the intervention period.

Nevertheless, a strength of our study was its long-term character (28 weeks, 56 sessions).

## 5. Conclusions

Our findings showed that a long-term training program (28 weeks) based on HIIT had a positive effect on anthropometric variables and CR, since it reduced OB and OW in schoolchildren. Hence, our results suggest that HIIT is a useful program for application during PA classes to improve health-related parameters in schoolchildren with OW and OB.

Further experimental investigations are needed to know the effect of increasing the number of PA sessions/week. Moreover, other factors such as daily diet should be controlled to better understand the effect of HIIT in schoolchildren, as this would help to produce a more sustainable change in the nutritional habits of schoolchildren.

## Figures and Tables

**Table 1:** Anthropometric data and CR before (pre-test) and after (post-test) intervention.

Variables	Groups	Mean (SD) Pre-Test	Mean (SD) Post-Test	*p*-Value (Time × Group)	Cohen’s d	Increase	*p*-Value
BMI (kg/m^2^)	EG CG *p*-value (group × time)	20.29 (2.81) 16.20 (0.86) <0.001	19.62 (2.75) 16.21 (1.26) <0.001	<0.001 0.507	0.240 0.009	0.66 (1.67) 0.08 (0.89)	<0.001
WC (cm)	EG CG *p*-value (group × time)	62.08 (5.48) 54.29 (2.78) <0.001	61.78 (6.56) 55.56 (4.34) <0.001	0.413 0.018	0.049 0.348	0.29 (3.95) 1.27 (3.99)	0.016
WHtR	EG CG *p*-value (group × time)	0.50 (0.03) 0.45 (0.02) <0.001	0.48 (0.04) 0.48 (0.30) 0.963	0.220 0.139	0.565 0.141	0.02 (0.03) 0.03 (0.30)	0.052
body fat (%)	EG CG *p*-value (group × time)	22.02 (5.54) 15.08 (3.46) <0.001	20.61 (5.47) 15.20 (3.55) <0.001	<0.001 0.675	0.256 0.034	1.41 (3.60) 0.08 (2.81)	<0.001
∑ 4 skinfold fat (mm)	EG CG *p*-value (group × time)	49.05 (16.18) 28.15 (7.12) <0.001	44.98 (16.50) 28.65 (8.25) <0.001	<0.001 0.421	0.249 0.064	4.06 (8.52) 0.50 (5.63)	<0.001
Systolic BP (mm Hg)	EG CG *p*-value (group × time)	101.28 (11.43) 95.85 (11.01) <0.001	102.06 (14.87) 98.78 (11.44) 0.027	0.406 0.026	0.058 0.260	0.78 (16.93) 2.93 (13.69)	0.179
Diastolic BP (mm Hg)	EG CG *p*-value (group × time)	60.85 (10.65) 59.70 (12.09) 0.295	60.45 (13.59) 59.72 (13.30) 0.638	0.662 0.944	0.032 0.001	−0.40 (16.97) 0.02 (17.90)	0.757
RHR (bpm)	EG CG *p*-value (group × time)	86.54 (12.58) 84.10 (12.31) 0.055	85.20 (11.37) 84.24 (12.25) 0.418	0.109 0.907	0.111 0.011	−1.34 (14.04) 0.14 (14.44)	0.306

BMI: body mass index; WC: waist circumference; WHtR: waist to height ratio; BP: blood pressure; RHR: resting heart rate; Bpm: beats per minute.

## Data Availability

Not applicable.

## Abbreviations

BF	Body Fat
BP	Blood pressure
BMI	Body mass index
CG	Control group
CR	Cardiometabolic risk
EG	Experimental group
HIIT	High intensity interval training
OB	Obesity
OW	Overweight
P	Percentile
PA	Physical activity
PE	Physical Education
PP	Percentage points
WC	Waist circumference
WFS	Waist for stature
WHtR	Waist to height ratio
